# Cinchonia Alkaloid

**Published:** 1879-06-20

**Authors:** T. S. Lallerstedt

**Affiliations:** Georgia


### *CINCHONIA ALKALOID.*

BY T. S. LALLERSTEDT, M.D., OF GEORGIA.

During the month of February, 1879, I received from Messrs. Powers & Weightman a hospital package each of cinchonia alkaloid and cinchonia mixture, for which I return my sincere thanks.

I have given it a thorough trial in my practice, and find that in 89 out of 100 cases it meets all the requirements of sulphates of quinine, cinchonidia or cinchonia, and besides it is almost tasteless, and produces no roaring in the head as quinine or cinchonidia, or the dryness of sulphate of cinchonia; and besides all this it is so much cheaper than either of the other three articles named.

I will give a few cases just as they occurred in my practice.

1st. Mr. W. C. Hook, 87 years of age, has been in poor health for nearly a year, having had a severe attack of remittent fever in April and May, 1878. On February 21st, 1879, I was called to see him, but being unable to go, I made inquiries about his case, and found he was having genuine shaking ague every afternoon, lasting from one to three hours with very high fever; ague coming on about 3 o'clock p. m.

I sent the following:

℞ Cinchon'a alkaloid.............................. grs 75

divide into 15 powders. Was directed to give five each day, and last was to be taken half hour before expected chill.

First day he only took 4, with light chill and no great deal of fever; second day, he took 5, the chill arrested. He has, up to this time, had no return of chill. I saw him in the month of April and he was still doing finely. In this case I gave cinchonia with no combination of any other medicines, as I was desirous of seeing if it would do. I generally combine opii pulv. comp. and capsicum in all cases of chill, and in almost all cases of fever, I combine opii pulv. compound, or opii pulv. alone.

2d. A lady was having chills. I sent her a quantity of cinchonia with direction for using it. It gave relief at once, and she has been doing well since.

I treated one case of intermittent pneumonia with it, with best of results, but it returned twice, on seventh day each time; but on the third attack was cured. I continued the use of medicine for a week after patient was up and about.

3d. Mrs. L. was attacked on Sunday night, March 16th, 1879, with remittent fever. I gave

Calomel...................................... grs viij
Aloes pulv.................................... grs ij M.

One powder as soon as fever began to cool. I gave cinchonia alkaloid grs. v, every two hours until four doses had been taken. During the height of fever I gave her tinc. gelsemii, gtts. 15 every three hours, and when fever began to cool gave cinchonia every three hours and continued gelsemii also, and wrapped head up in cold cloths. Fever gave way by Wednesday morning, 19th March, and no return.

I would ask my country brethren to give this alkaloid a fair trial. We who furnish our medicines and have to wait several months or years for our money must endeavor to find good, reliable and cheap medicines. We cannot afford to make large bills with our patrons. Quick cures and small bills I find to pay better than slow cures and large bills.

---

**Thermoscope and Hydroscope.**—A novel thermoscope and hydroscope, the invention of Col. Aristide Gerard, has recently been patented both in this country and in Europe, and is controlled by the Automatic Safety Company, of No. 40 Charles street, New Orleans, La. This invention is designed for the speedy detection of abnormal heat or water in steamers and other vessels, and is said to be very effective. —*Scientific American.*

